# A case of tetanus treated with Kampo medicines such as Kakkonto and Shakuyakukanzoto

**DOI:** 10.1002/ams2.254

**Published:** 2016-12-01

**Authors:** Hajime Nakae, Yuri Saito, Manabu Okuyama, Toshiko Igarashi

**Affiliations:** ^1^ Department of Emergency and Critical Care Medicine Akita University Graduate School of Medicine Akita Japan

**Keywords:** Generalized tetanus, kakkonto, shakuyakukanzoto, traditional Japanese herbal medicine, trismus

## Abstract

**Case:**

A 74‐year‐old man developed tetanus 3 days after working with cow and poultry manure. Kakkonto and shakuyakukanzoto, traditional Japanese herbal medicines that are effective for the relief of pain primarily related to muscle contraction, were given to control the trismus and painful contracture of the neck. Generalized convulsions were controlled without the use of muscle relaxants.

**Outcome:**

After 30 days, the patient was discharged from the hospital without any sequelae.

**Conclusion:**

Kakkonto and shakuyakukanzoto may be useful for the control of muscle spasms resulting from generalized tetanus.

## Introduction

Tetanus is an infection characterized by muscle spasms and various types of autonomic hyperactivity. It is caused by *Clostridium tetani*, which produces tetanospasmin. In Japan, approximately 100 patients are infected with tetanus annually. Trismus is a well‐known symptom, and muscle relaxants are often necessary to relieve generalized convulsions. As the use of muscle relaxants may cause muscle atrophy, difficult expectoration of sputum, or ventilator‐associated pneumonia, their long‐term use should be avoided.^1^ In Kampo medicine, kakkonto (KT) and shakuyakukanzoto (SKT) are effective for the relief of pain primarily related to muscle contraction. We previously reported that KT and SKT were used for generalized convulsions caused by tetanus and successfully treated without the use of a muscle relaxant in two cases.^2^, ^3^ We report another case of tetanus herein; in our experience, Kampo treatment using KT and SKT can be an additional therapy for tetanus.

## Case

**Figure 1:**
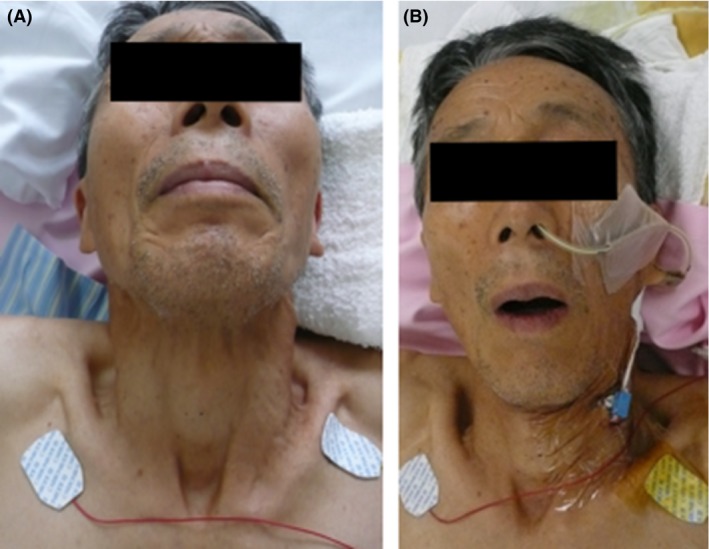
Facial appearance of a 74‐year‐old man with tetanus. A, Facial appearance on arrival at hospital; trismus, contraction of the sternocleidomastoid, and stiffness in the neck are observed in the upper body. B, Appearance of the face 30 min after Kampo extracts were given. The painful contracture of the neck and trismus were improved 30 min after 7.5 g kakkonto and 7.5 g shakuyakukanzoto were given simultaneously.

**Figure 2:**
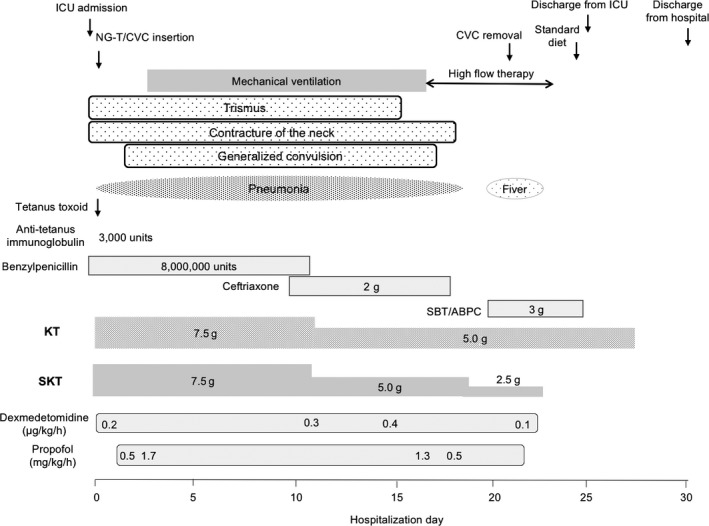
Clinical course of a 74‐year‐old man with tetanus. CVC, central venous catheter; ICU, intensive care unit; KT, kakkonto; NG‐T, nasogastric tube; SBT/ABPC, sulbactam sodium ampicillin sodium; SKT, shakuyakukanzoto.

A 74‐year‐old man with a history of diabetes mellitus and chronic obstructive pulmonary disease suffered cervicalgia and a stiff shoulder after engaging in farm work. He was a chicken farmer and produced a fertilizer that utilized cow and poultry manure. He went to a nearby clinic 2 days after the onset of symptoms as he experienced difficulty swallowing. Tetanus was strongly suspected from his clinical condition, and he was transferred to the emergency room 3 days after the initial symptoms began. His vital signs were as follows: Glasgow Coma Scale score, 15 (E4V5M6); blood pressure, 144/63 mmHg; heart rate, 79 b.p.m.; respiratory rate, 24 breaths/min; and body temperature, 36.5°C. The level of oxygen saturation showed 96%. Painful contracture of the neck was observed. Trismus, risus sardonicus, and strong muscle spasms in the platysma and sternocleidomastoideus were observed (Fig. 1). Laboratory tests revealed slight inflammation and rhabdomyolysis (white blood cell count, 12,600 cells/mm^3^; C‐reactive protein, 0.04 mg/dL; lactase dehydrogenase, 276 U/L; creatine kinase, 340 IU/L; blood urea nitrogen, 25.5 mg/dL). A computed tomography scan showed emphysematous bulla in the lung and patchy pneumonia in the right upper lobe. Tetanus was clinically diagnosed and he was admitted into the intensive care unit (Fig. 2). Polyethylene glycol‐treated human anti‐tetanus immunoglobulin (3,000 units) was injected and benzylpenicillin potassium (8,000,000 units/day) was administered. Both KT (7.5 g/day) and SKT (7.5 g/day) were given by nasogastric tube to release muscle tension. One to three packages of each Kampo extract was dissolved in tap water (20 mL). Trismus and generalized convulsions were temporarily improved 30 min after treatment with KT and SKT (Fig. 1B). Fluctuation of systolic blood pressure (100–170 mmHg) was observed for the first 3 days of hospitalization. It was most severe on day 1 (70–180 mmHg). On day 3 of hospitalization, mechanical ventilation was introduced to treat the complications of pneumonia. Blood gas analysis revealed pH 7.47, PaO_2_ 87 mmHg, PaCO_2_ 41 mmHg, base excess (BE) 5.6 mEq/L (F_I_O_2_ 0.6, synchronized intermittent mandatory ventilaton (SIMV) 13/min, pressure control (PC) 10 cmH_2_O, pressure support (PS) 8 cmH_2_O, positive end‐expiratory pressure (PEEP) 4 cmH_2_O). After 11 days of hospitalization, doses of KT and SKT were reduced to 5.0 g/day, as the tetanic convulsions were controlled without the use of muscle relaxants. After 17 days of hospitalization, the patient was weaned from the ventilator. The patient was discharged from the intensive care unit after 25 days; after 27 days, treatment with all Kampo medicines was terminated. After 30 days of hospitalization, he was discharged from the hospital without sequelae.

The blood culture at the time of the patient's admission showed negative results; however, on day 4 of hospitalization, *Klebsiella oxytoca* was detected from the sputum culture.

## Discussion

Treatment of tetanus is broadly divided into the following four aspects: (i) prevention of the production of exotoxin, (ii) neutralization of exotoxin, (iii) control of myospasms, (iv) the alleviation of autonomic hyperactivity. In our case, benzylpenicillin potassium was used. For the neutralization of exotoxin, an antitetanic human immunoglobulin was used.

Tetanic convulsion, associated with the risk of developing respiratory failure, requires strict management. Propofol has been reported to be effective. However, because convulsions cannot be controlled by sedatives in some severe cases, it may be necessary to use muscle relaxants in combination with sedatives. In recent years, magnesium sulfate has also been used for prevention of autonomic hyperactivity; however, magnesium sulfate is reported to cause adverse reactions such as central nervous system disorders and hypocalcemia.^4^

Shakuyakukanzoto is a Kampo medicine used for the treatment of pain associated with sudden myospasms. Regarding the effects on peripheral muscle relaxation, Kimura *et al*.^5^ reported that SKT blocks neuromuscular synapses to relax skeletal muscles. Moreover, Kurosawa *et al*. reported that SKT has a relaxation effect on gastrointestinal smooth muscles.^6^ In addition, although SKT has an inhibitory effect on myospasms, it is reported that this medicine does not affect normal physiological twitching.^7^, ^8^ Presumably, SKT may relieve muscle ischemia caused by muscle contractions and, subsequently, the myalgia associated with ischemia. Furthermore, glycyrrhizin, a major component of licorice, also has an inhibitory effect on the production of prostaglandins and exerts an analgesic effect.^9^ In our case, the concomitant use of SKT allowed us to reduce the dosage of propofol and to avoid the use of muscle relaxants. For the treatment of autonomic hyperactivity due to tetanus, the use of central α_2_ receptor agonists, such as clonidine hydrochloride, methyldopa, and dexmedetomidine, has been reported.^10^ Shakuyakukanzoto is also reported to exert a central analgesic effect by activating the descending noradrenergic system (α_2_ receptors) in the spinal cord.^11^ Girgin *et al*. reported that treatment with dexmedetomidine did not fully control muscle spasms but decreased their frequency and severity, and reduced the use of sedative, analgesic, and muscle relaxant drugs to control muscle spasms and cardiovascular instability.^12^ In our case, the use of dexmedetomidine in combination with Kampo medicine may have contributed to stable hemodynamics and analgesia.

Kakkonto is prepared by adding pueraria root and ephedra to keishito, which is a composite of cinnamon bark, ginger, jujube, peony root, and licorice. Kakkonto is used to treat anhidrosis and stiffness of the neck and back. The pueraria root contains flavonoids, such as daidzein and isoflavone, and has a papaverine‐like antispasmodic effect. Ephedra contains ephedrine and exerts an adrenaline‐like effect. In addition, ephedra has been revealed to have anti‐inflammatory, analgesic, and antispasmodic effects. The cinnamyl compound, which is abundantly contained in cinnamon bark, inhibits the overproduction of interleukin‐1α and is also reported to alleviate viral pneumonia.

When KT and SKT are used concomitantly, attention should be paid to hypokalemia caused by pseudoaldosteronism, which may result from an adverse reaction to licorice. In our case, although licorice was given at a daily dose of up to 8 g, the serum potassium levels remained within the normal range, showing no marked fluctuation.

We have previously reported two cases in which the application of KT and SKT for the treatment of tetanus enabled us to avoid the use of mechanical ventilation and muscle relaxants.^2^, ^3^ In the present case, the patient complained of trismus and painful contracture of the neck. Because we assumed that KT alone was insufficient to prevent generalized tonic convulsions, SKT was used concomitantly. Although the patient was placed on mechanical ventilation due to the concomitant occurrence of pneumonia, symptoms were relieved without the use of muscle relaxants. To ensure prevention of myospasms, the concomitant use of muscle relaxants is inevitable in severe cases. However, as for dantrolene sodium, adverse effects such as central nervous system damage and liver dysfunction are well known. In patients who develop respiratory depression and have a history of chronic obstructive pulmonary disease, like our patient, management with mechanical ventilation may be prolonged. In the present case, the patient was weaned from mechanical ventilation after 14 days. This is greatly attributed to the fact that we were able to follow up without the use of muscle relaxants. In addition, our patient may have responded to the Kampo medicine with a resulting antispasmodic effect.

## Conflict of Interest

Hajime Nakae has received lecture fees from Tsumura & Co. The other authors have no conflict of interest.
